# Supplementary material for: Structural multi-colour invisible inks with submicron 4D printing of shape memory polymers
Source: Nat Commun. 2021 Jan 4;12:112. doi: 10.1038/s41467-020-20300-2 (PMC7782480; doi:10.1038/s41467-020-20300-2)
Supplement: Supplementary file 1 — Supplementary Information [file 41467_2020_20300_MOESM1_ESM.pdf]

Supplementary Information

Structural Multi-Colour Invisible Inks with Submicron 4D Printing of Shape Memory Polymers

Wang Zhang[1], Hao Wang[1*], Hongtao Wang[1], John You En Chan[1], Hailong Liu[1, 2], Biao Zhang[3], Yuan-Fang Zhang[4], Komal Agarwal[1], Xiaolong Yang[5], Anupama Sargur Ranganath[1], Hong Yee Low[1], Qi Ge[6], Joel K.W. Yang[1,2*]

[1]Engineering Product Development, Singapore University of Technology and Design, Singapore 487372, Singapore. [2]Nanofabrication Department, Institute of Materials Research and Engineering, Singapore 138634, Singapore. [3]Frontiers Science Center for Flexible Electronics, Xi'an Institute of Flexible Electronics (IFE) and Xi'an Institute of Biomedical Materials & Engineering (IBME), Northwestern Polytechnical University, 127 West Youyi Road, Xi'an 710072, China. [4]Digital Manufacturing and Design Centre, Singapore University of Technology and Design, Singapore 487372, Singapore. [5]National Key Laboratory of Science and Technology on Helicopter Transmission, Nanjing University of Aeronautics and Astronautics, Nanjing 210016, China. [6]Department of Mechanical and Energy Engineering, Southern University of Science and Technology, Shenzhen 518055, China.

*email: hao_wang@sutd.edu.sg; joel_yang@sutd.edu.sg

1. Chemical compositions of the photoresist

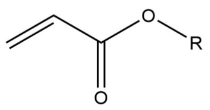

VERO

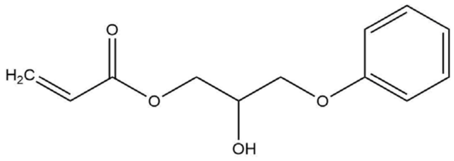

2-hydroxy-3-phenoxypropyl acrylate

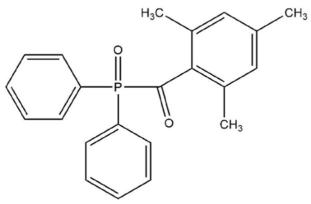

Diphenyl(2,4,6-trimethylbenzoyl)phosphine oxide

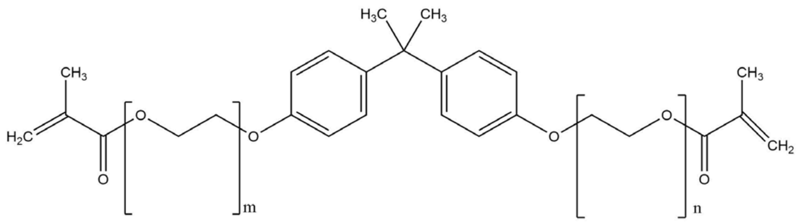

Bisphenol A ethoxylate dimethacrylate

Supplementary Figure 1. Chemical structures of the components in the customized photoresist.

2. Materials characterization

To develop a suitable resist for additive manufacturing of shape memory polymer, one needs to satisfy several criteria simultaneously: (1) sufficient viscosity for patterning with our TPL system, (2) a suitable T_g, e.g., above room temperature, and (3) sufficient deformability to avoid irreversible damage during the programming process as described in Fig. 1. To meet these demands, we cured macroscopic films (0.5mm thick) of the resist in ultraviolet light to perform mechanical tests. Results are shown in Supplementary Fig. 2a-d. As the temperature increases, the storage modulus decreases for all concentrations due to the viscoelasticity of the SMP (Supplementary Fig. 2a). At the same temperature, the storage modulus decreases as the increase of the concentration of the elastomer, which has a much smaller elasticity modulus than Vero Clear. Supplementary Fig. 2b plots tan δ, i.e., the ratio of loss modulus (corresponding to energy dissipation) and storage modulus, as a function of temperature. The peaks of these plots correspond to the glass transition temperature of the particular composition of Vero Clear to elastomer ratio. A single tan delta peak indicates that the blend of elastomer and Vero Clear co-cured without phase separation. The T_g for pure Vero Clear is ~65 °C and ~17 °C for pure elastomer. As the concentration of the elastomer increases from 10% to 50%, T_g decreases from 54 °C to 29 °C. Varying the ratio of Vero Clear to elastomer thus allows for prints that respond to a range of temperatures. For convenience, we chose a T_g of ~40 °C, i.e., above room temperature for the programmed structures to be maintainable without external load at room temperature.

Supplementary Fig. 2c presents the stress-strain curves of the polymer blends above T_g. The temperature was set to 20 °C above the corresponding T_g for each composition. As the blend of Vero Clear and the elastomers is homogenous as shown by the tan delta results, expectedly, the strain at failure increases from 34% to 85% (Supplementary Fig. 2c inset) with increasing elastomer content. For the interest of this study, the mass ratio of 7:3 of Vero Clear and elastomer was chosen which has a strain at failure of ~50%. It should be noted that the mechanical tests in Supplementary Fig. 2a-c were conducted by UV cured macroscopic films of 0.5 mm and the patterned film by the TPL process is in the submicrometer range. The mechanical properties of the TPL and UV patterned structures may be different due to the difference of degree of conversion[1], alignment of the polymer chains[2], and size effect[3]. Micro and Nanoscale DMA[4], compression[5] and tension[6] need to be conducted to directly determine the mechanical properties. Nonetheless, the test data provided here can be used as a rough guide to study the trends of mechanical properties as a function of chemical compositions. We used

UV light to post cure the printed samples as mentioned in the methods section of the manuscript to minimize the difference of mechanical properties between UV and TPL fabricated samples.

In the Dip-in Laser Lithography (DiLL) configuration of Nanoscribe, the viscosity of the photoresist should be controlled: A viscosity that is too low would result in the resist flowing away (unless gaskets are used) as the sample stage moves during printing. A viscosity that is too high could reduce the print resolution by suppressing the diffusion of oxygen inside the resist, which acts as quencher of the photopolymerization process[7]. The developed SMP photoresists in our work have viscosities between 140 to 220 mPa·s (as shown in Supplementary Fig. 2d), which is about 10 times less viscous than the commercial photoresist IP-Dip (2420 mPa·s according to the official datasheet). Nonetheless, we were able to expose and pattern high resolution structures without problem, indicating a suitable viscosity range of the resist within the TPL system.

To examine the resolution of this resist, we exposed a series of single-pixel lines on a base layer (~2μm thick), used to aid adhesion. Supplementary Fig. 2e-f show scanning electron micrographs (SEM) of patterns formed in a resist with a 7:3 mass ratio composition of Vero Clear to elastomer. The linewidth decreases as expected with decreasing laser power and increasing write speed (Supplementary Fig. 2e). The polymerization threshold of 30 mW was determined at a reasonable write speed of ~1.1mm/s resulting in a linewidth of ~280 nm. This write speed is an order of magnitude slower than that for the commercial TPL photoresist IP-Dip[8] , which may be attributed to lower reaction speeds of both the initiator and monomer. Though the linewidth can be further reduced by lowering the laser power, the resultant structures tend to collapse due to poor mechanical strength[9]. Hence, the lowest laser power used in this work was 30 mW. To determine the resolution limit, we fabricated lines with different pitches with a fixed write speed of 1.1mm/s while varying the laser power, as shown in Supplementary Fig. 2f. A conservative minimum resolvable pitch was determined to be 600 nm (i.e., 300 nm half pitch). Gaps between lines at 400 nm pitch were not well defined.

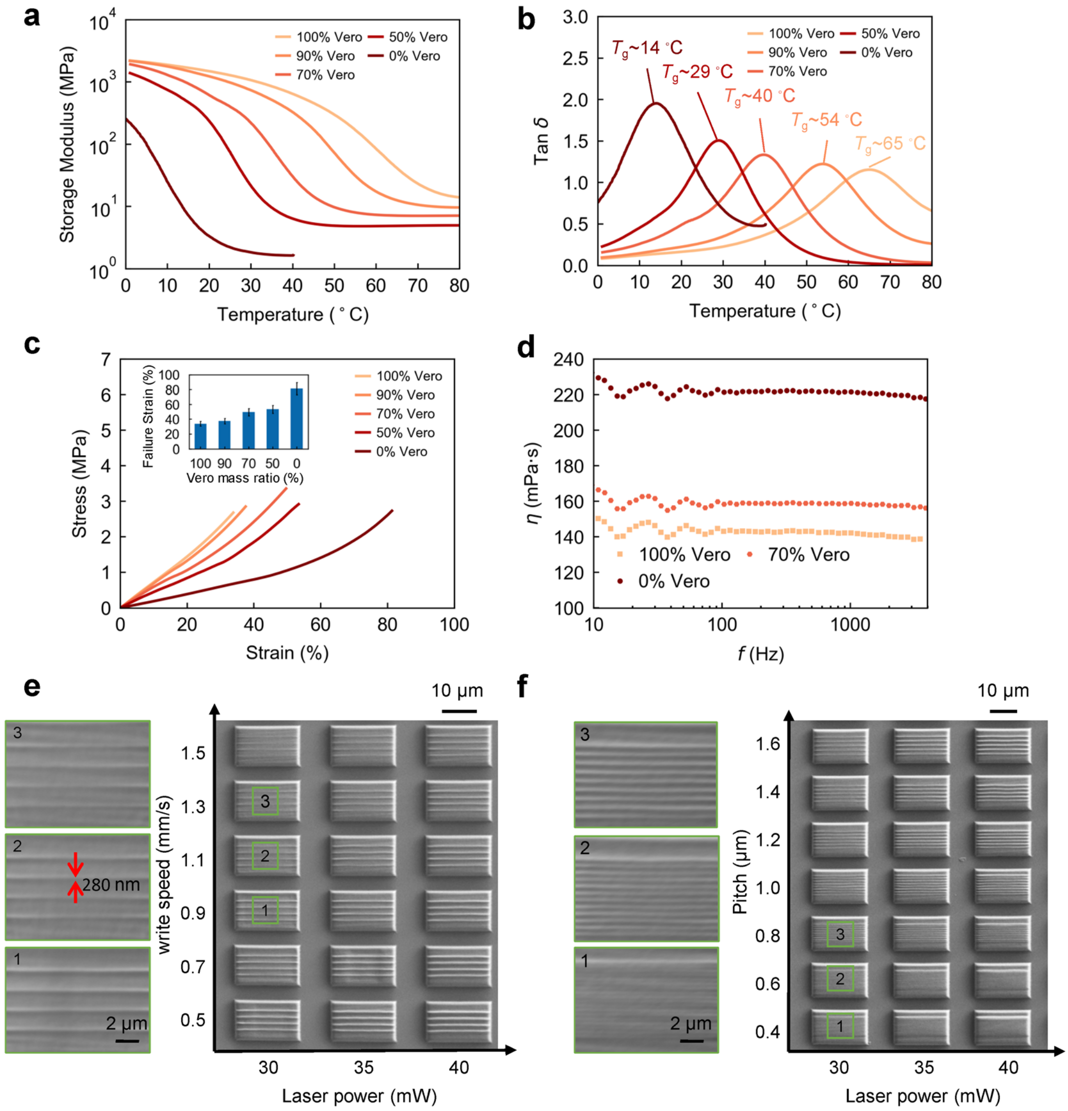

Supplementary Figure 2. Mechanical characterization for different mass ratios of Vero Clear to elastomer in the photoresist. (a) Storage modulus as a function of temperature. (b) Plot of tan δ values versus temperature. (c) Uniaxial tensile tests at high temperature at 20 °C above the glass transition temperature (T_g). (inset) Failure strain at different Vero Clear mass ratios. (d) Photoresist viscosity tests for pure Vero Clear, pure elastomer and 70% Vero Clear, respectively. (e) Left: High-magnification Scanning Electron Micrographs (SEM) of single-pixel lines fabricated with laser power of 30mW and write speed of 0.9mm/s, 1.1 mm/s and 1.3 mm/s respectively; Right: SEM images of lines fabricated at different laser powers and write speeds to determine crosslinking threshold. (f) Left: High-magnification SEM images of lines fabricated with laser power of 30 mW, write speed of 1.1 mm/s and pitches 0.4 μm, 0.6 μm and 0.8 μm respectively; Right: SEM images of lines fabricated with a fixed write speed of 1.1mm/s and different laser powers to determine print resolution.

3. Determination of laser power and write speed to fabricate the grid structures

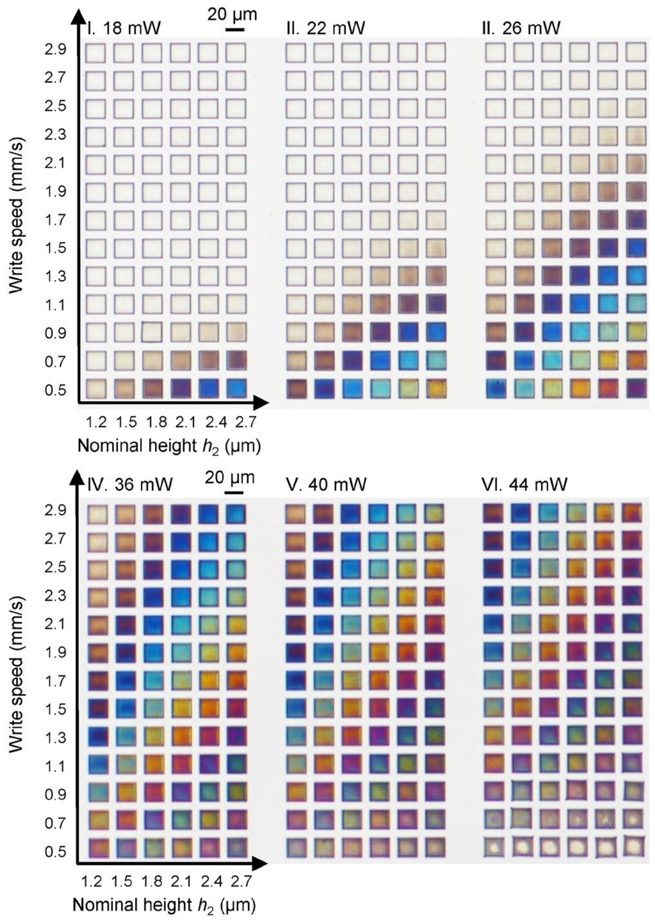

Supplementary Figure 3. Optical transmittance micrographs of a printed colour palette for a constant pitch of 2 µm but varying write speed and nominal height h_2 for a wide range of laser power.

Relatively narrow ranges of laser power (30-35 mW) and write speed (0.5-1.4 mm/s) were chosen for Fig. 2a in the manuscript. Decreasing the laser power results in some colourless structures in the higher write speed regions (Supplementary Fig. 3 I-III), which can be resulted from lack of polymerization ratio and mechanical strength of the grids above the base to survive either during printing or developing. While increasing the laser power can also generate different colours except the ones with low write speeds (0.5-0.7 mm/s) and high laser power (44 mW) due to over exposure (Supplementary Fig. 3 IV-VI). We also noted that either increasing or decreasing the laser power would not generate new colours significantly different from the ones we have shown in Fig. 2a. Hence, relatively narrow ranges of laser power (30-35 mW) and write speed (0.5-1.4 mm/s) were chosen as "safe" parameters in Fig. 2a. In other words, the process window is not particularly large. The observable colour changes within the narrow range of power shows that.

4. Fabrication parameters

Different structures in the manuscript were fabricated with different parameters. See Table S1 for the fabrication parameters.

Supplementary Table 1. Fabrication parameters*

Fig.s	Laser power (mW)	Write speed (mm/s)	Pitching (μm)	Hatching (μm)	Number of grid layers
Supplementary Fig. 2e (Bottom surface)	35	2	0.2	0.4	5
Supplementary Fig. 2e (Up grids)	30-45	0.5-1.5	2	0.3	1
Supplementary Fig. 2f (Bottom surface)	35	2	0.2	0.4	5
Supplementary Fig. 2f (Up grids)	30-45	1.1	0.4-2	0.3	1
Fig. 2a (Bottom surface)	35	2	0.2	0.4	10
Fig. 2a (Up grids)	30-35	0.5-1.4	2	0.3	5-10
Supplementary Fig. 5 (Bottom surface)	35	2	0.2	0.4	10
Supplementary Fig. 5 (Up grids)	30	0.6	2	0.3	7
Fig. 4a (Bottom surface)	35	2	0.2	0.4	10
Fig. 4a (Up grids)	30	0.6-1.1	2	0.3	7
Fig. 4d-f (Bottom surface)	35	2	0.2	0.4	10
Fig. 4d-f (Up grids)	32.5	0.8	2	0.3	7
Fig. 4g (Bottom surface)	35	2	0.2	0.4	10
Fig. 4g (Up grids)	30	0.5-1.2	2	0.3	4-9

(*The first layer of grids was printed at the same vertical coordinate as the last layer of the base surface to increase bonding between the grids and the base surface.)

5. Calculated colour coordinates of Fig. 2aI in the CIE 1931 chromaticity diagram

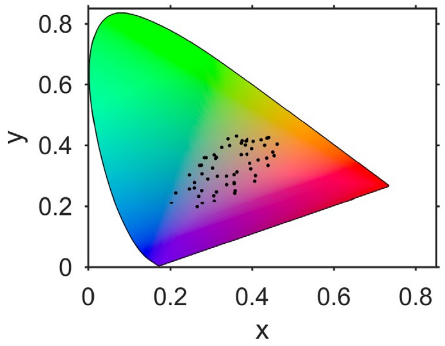

Supplementary Figure 4. Calculated colour coordinates of Fig. 2aI mapped onto the CIE 1931 chromaticity diagram, providing an indication of the gamut achieved from the grid structures.

6. Effect of pitch w_2 on the spectra

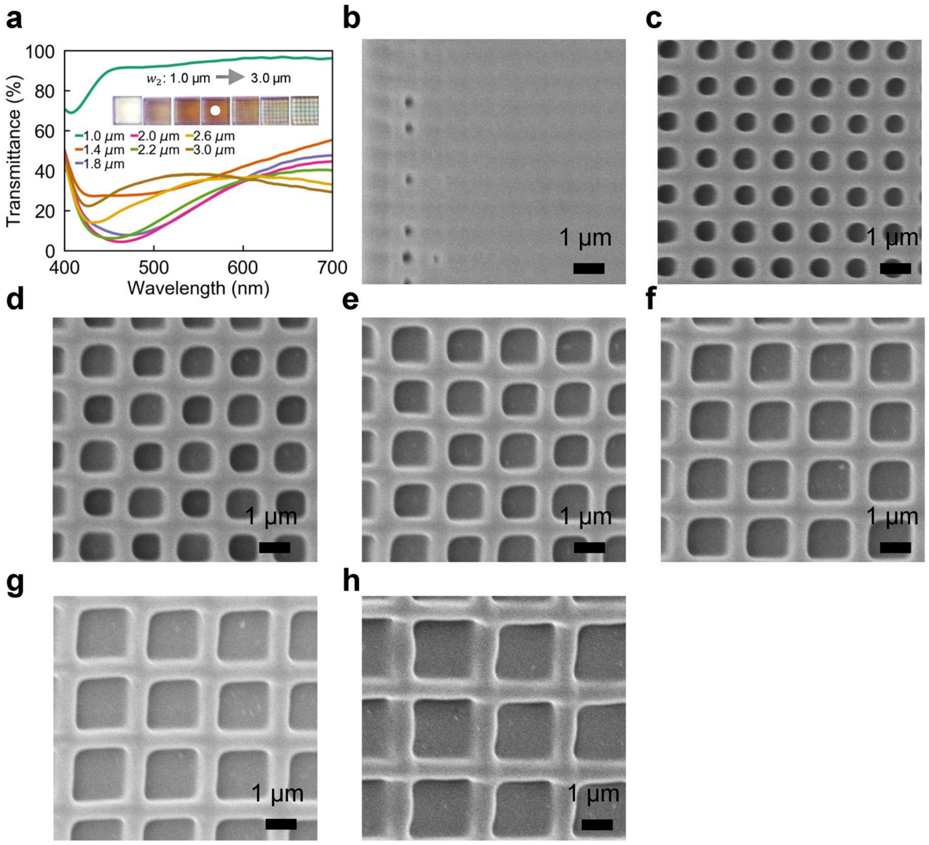

Supplementary Figure 5. (a) A comparison of transmittance spectra for different pitches w_2. (b)-(h) SEM images of grid structures with pitches of 1 μm, 1.4 μm, 1.8 μm, 2.0 μm, 2.2 μm, 2.6 μm, 3.0 μm, respectively.

7. SEM images of grid structures fabricated with different laser power

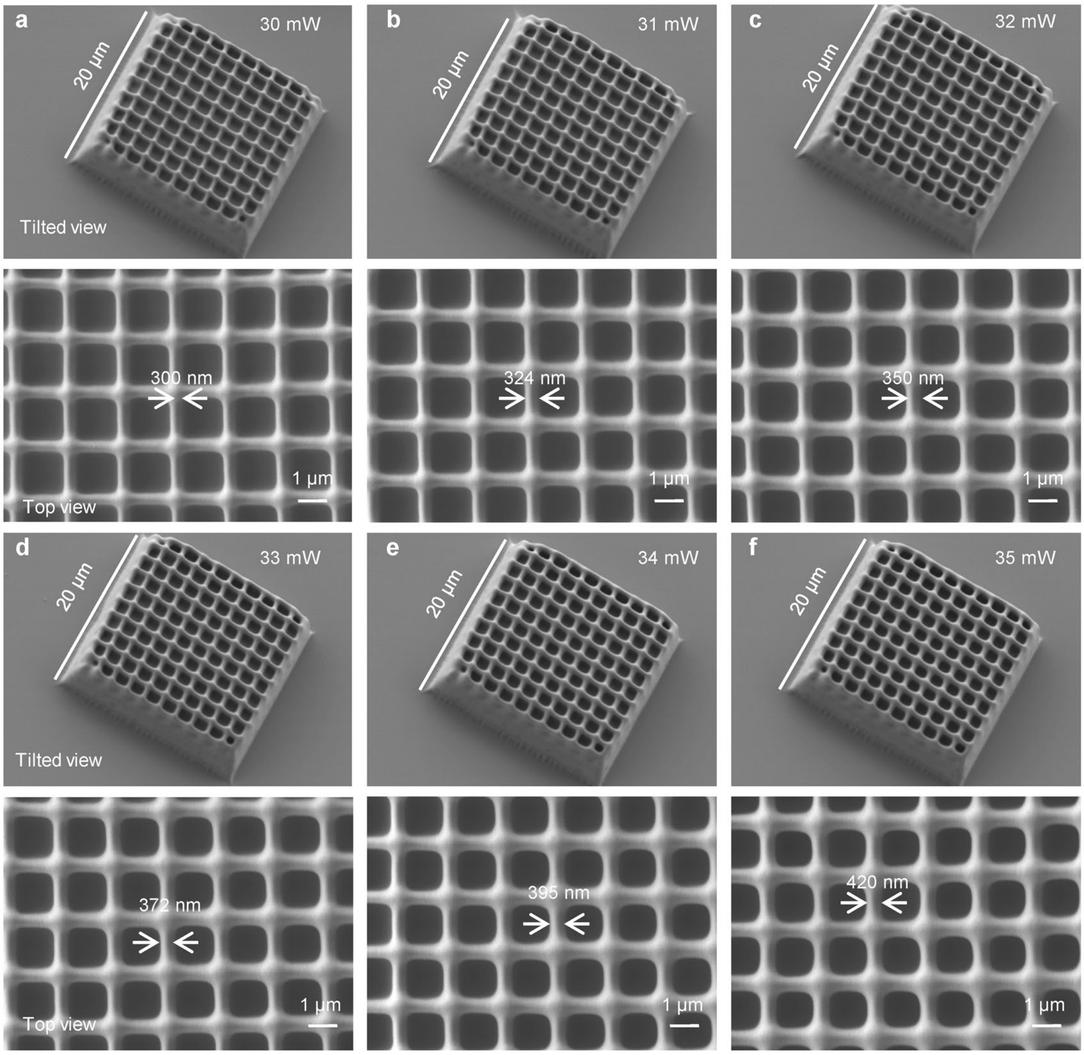

Supplementary Figure 6. SEM images of grid structures fabricated with fixed write speed 1mm/s and nominal height $w_2 = 1.8$ µm while varying the laser power from 30 mW to 35 mW.

8. Determining of the refractive index used in the FDTD simulation

To measure the refractive index of the photoresist, a wafer substrate was first washed by IPA and then baked on a hotplate at 130 °C for 10 mins. Then the resist was spin coated on the substrate with a speed of 7000 rpm for 1 min. Then the substate was baked again on the hotplate 130 °C for 3 mins.

The ellipsometry angles Δ and Ψ were measured by an EP4 ellipsometer (ACCURION, Germany). The measured data were fitted by the Cauchy dispersion function as shown in Eq. (S1) and Eq. (S2), where A, B, C, A_1, B_1, C_1 are parameters for the Cauchy dispersion which can be determined by fitting the measured ellipsometric angles Δ and Ψ. n and k are refractive index and extinction coefficient of the material, respectively. The fitting process was done by the Levenberg-Marquardt algorithm in the EP4 model software.

$$n(\lambda) = A + \frac{B}{\lambda^2} + \frac{C}{\lambda^4} \quad \text{(S1)}$$

$$k(\lambda) = A_1 + \frac{B_1}{\lambda^2} + \frac{C_1}{\lambda^4} \quad \text{(S2)}$$

The fitted parameters are given in Table S2

Supplementary Table 2. Cauchy dispersion parameters for the photoresist

A	B	C	A_1	B_1	C_1
1.489	2998	4.317*e8	0	0	0

After fitting the dispersion function, the refractive index n and k as functions of wavelength are obtained and plotted in Supplementary Fig. 7.

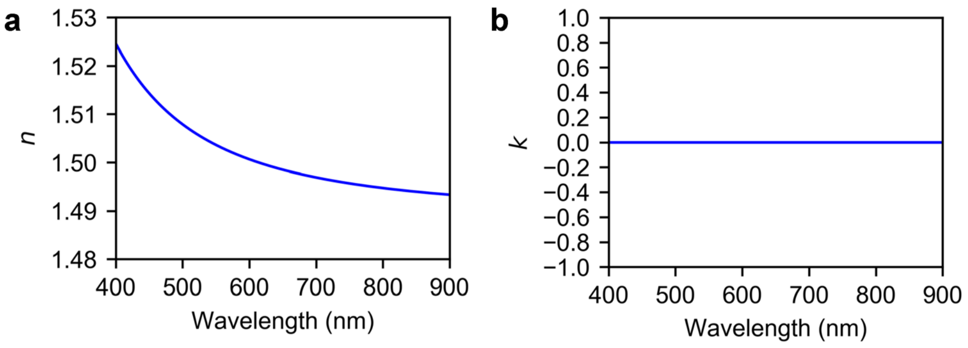

Supplementary Figure 7. Determining of the refractive index. (a) Fitted refractive index n; (b) Fitted extinction coefficient k.

9. A comparison of the measured and simulated spectra

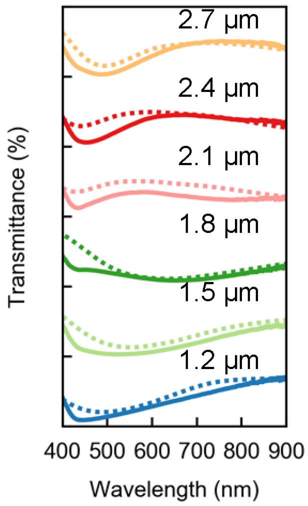

Supplementary Figure 8. A comparison of the measured and simulated spectra.

10. Relation between line width w_1 and dip position

As the increase of line width w_1, the effective index $R_e = \sqrt{\varepsilon_e}$ increases because the volume fraction of the polymer to air increases. The effective permittivity ε_e can be calculated by the modified Maxwell Garnett effective medium model[10],

$$\varepsilon_e = \varepsilon_a \frac{\varepsilon_p (1+2f) + \varepsilon_a (2-2f)}{\varepsilon_p (1-f) + \varepsilon_a (2+f)} \tag{S3}$$

where ε_p and ε_a are the permittivities of polymer and air respectively. $\varepsilon_p = n_p^2$ and $\varepsilon_a = n_a^2$, where n_p and n_a are the refractive indexes of polymer and air, respectively. n_p can be obtained by the fitted data in Supplementary Fig. 7a, and n_a=1.003. The volume fraction of polymer can be calculated using the geometry relation as shown in Supplementary Fig. 8a, and $f = \frac{w_2^2 - (w_2 - w_1)^2}{w_2^2}$ .The dip position λ can be obtained from the simulated data in Fig. 3f. Using the above data and formulas, the dip position and effective index as a function of line width can be obtained by the Least square polynomial fit as shown in Supplementary Fig. 8b. Both λ and n_e show strong linear relation with w_1, from which we can derive a linear relation between λ and n_e as $\lambda = 1500 n_e - 1210.5$. This relation is analogous to the relation between λ and n in diffraction grating formula $\lambda = \frac{dn \sin\theta_m}{m} + C$, where d is the grating period, θ_m is the angle between the diffracted ray and the grating's normal vector, C is a constant to be fitted.

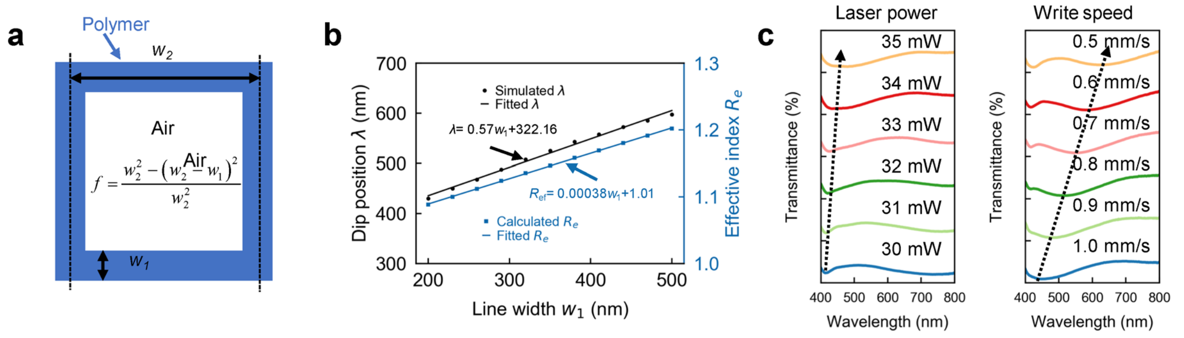

Supplementary Figure 9. (a) Determining of polymer volume fraction. (b) Dip position and effective index as a function of linewidth. (c) Measured spectra with the variation of fabricating laser power and write speed. (Left spectra were measured from structures in Fig. 2a with fixed write speed of 1 mm/s, nominal height h_2 of 1.8 μm; Right spectra were measured from structures in Fig. 2a with fixed laser power of 30 mW, nominal height h_2 of 2.7 μm).

11. The programming progress by hand

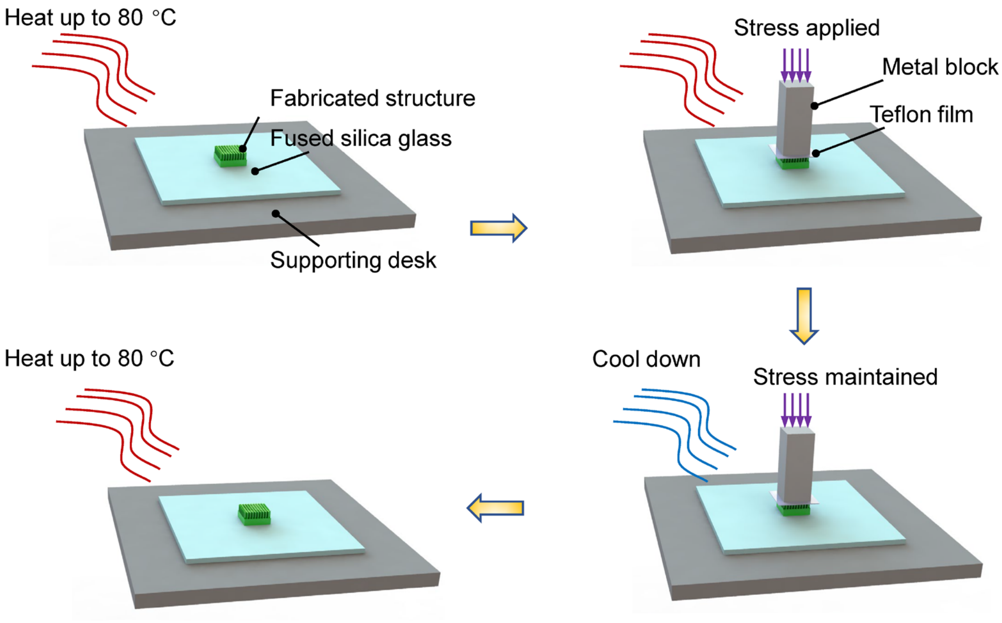

Supplementary Figure 10. Schematic of the 4D programming process. The structure was first heat up to 80 °C by a heat gun. The temperature was monitored by a thermometer. Then a stress of ~500kPa* was applied through a metal block. A layer of Teflon film was put between the structure and the block to avoid contamination. The heat gun was removed afterward, and the structure was cooled down to room temperature with the stress maintained. Finally, the stress was removed, and the structure was heat up to 80 °C to recover. (*The stress was estimated by the following process: A force applied by thumb was estimated to be ~50 N based on Ref.[11]; The contact area of the metal surface and the support was 1cm×1cm; The pressure $P \approx \frac{50\text{N}}{1\text{cm} \times 1\text{cm}} = 500\text{kPa}$).

12. Programming process by nanoimprint

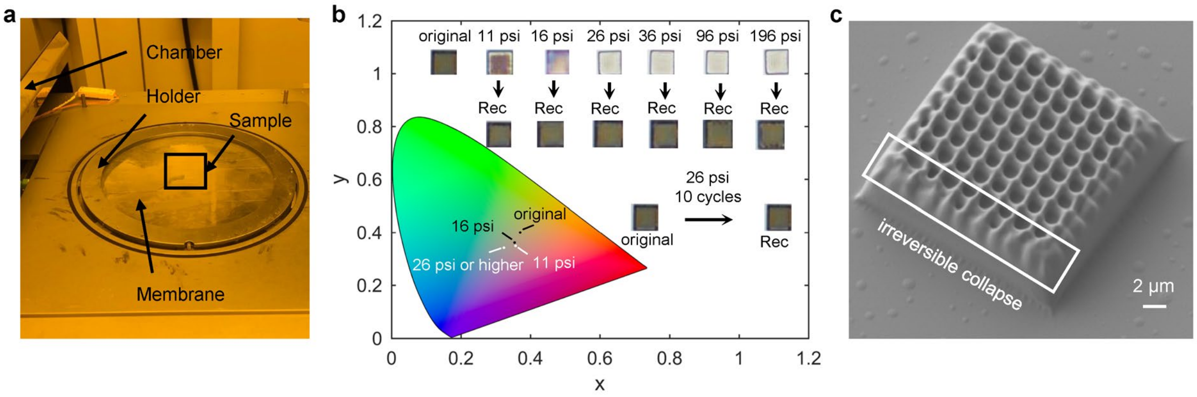

Supplementary Figure 11. Programming process by nanoimprint. (a) A photo of the setup of the Nanonex NX-2006 machine; (b) Change of colour as a function of applied pressure (Rec represents Recovered). (c) A recovered structure after 196 psi pressure applied in the programming process.

To quantify the influence of the stress on the programming process, we performed an additional programming process using a Nanonex NX-2006 machine (Supplementary Fig. 11a). In this set up as shown in Supplementary Fig. 11a, the structures printed on a glass substrate was put between two membranes fixed by the holder. A layer of Teflon film was put between the glass and the upper membrane to avoid contamination during process. After this setup, the holder was put into the chamber where the pressure and temperature can be controlled. Inside the chamber, the space between the membranes was first pumped to vacuum, then the sample was heated up to 50 °C, and then different pressure was applied to the membranes for 30 s. With the pressure maintained, the sample was cooled down to 22 °C (room temperature) to get the deformed configuration. The recovery process was induced by heating the sample to 80 °C by a heat gun. Note that here 50 °C (~10 °C above T_g) was chosen as the elevated temperature to apply stress, which is 30 °C lower than that in the programming process by hand in Supplementary Information part 11. This is because the whole programming process by this Nanonex NX-2006 machine was ~ 5 mins, while the whole programming process by hand was only ~30 s, and we found that it is safer to use a lower programming temperature (50 °C) to avoid irreversible deformation for a relatively long loading and unloading time.

Using this programming process, a structure was programmed under different loads. As the applied stress increases to 26 psi (~179 kPa) or higher, the structure turned to colourless (Supplementary Fig. 11b). While a high stress of up to 196 psi (~1351 kPa) leads to the irreversible collapse around the edges (Supplementary Fig. 11c). Note that in situ micron and

nanoscale compression experiments[5] at controlled temperatures will need to add to directly determine the relation between stress and strain during deformation of the structure. Based on this programming process, the repeatability of the shape memory process was further checked, and no obvious change of colour was observed after 10 programming cycles (Supplementary Fig. 11b). To further increase the number of program-recovery cycles (e.g., beyond 1000 cycles) the structure can stand, some specific chemicals such as monomers with strong π -π interactions and massive chain entanglements[12] should be considered in future studies.

13. SEM images of the painting

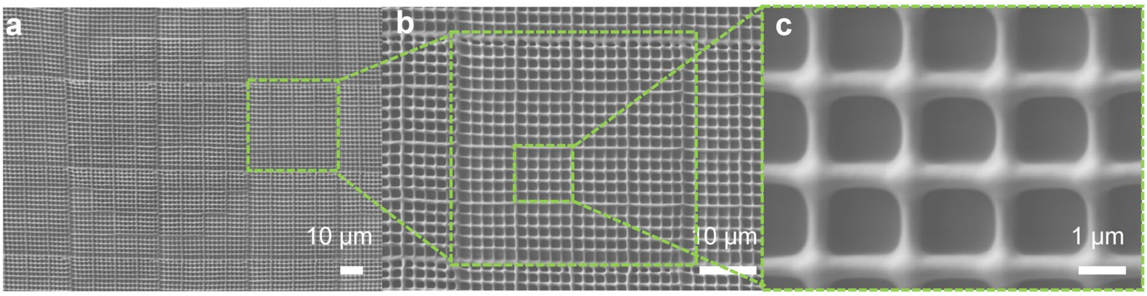

Supplementary Figure 12. (a)-(c) SEM images of the painting.

Supplementary References

1 Jiang, L. J. *et al.* Two-photon polymerization: investigation of chemical and mechanical properties of resins using Raman microspectroscopy. *Opt. Lett.* **39**, 3034-3037 (2014).

2 Zhao, X. *et al.* Bioinspired ultra-stretchable and anti-freezing conductive hydrogel fibers with ordered and reversible polymer chain alignment. *Nat. Commun.* **9**, 1-8 (2018).

3 Ushiba, S. *et al.* Size dependent nanomechanics of coil spring shaped polymer nanowires. *Sci. Rep.* **5**, 17152 (2015).

4 Chakravartula, A. & Komvopoulos, K. Viscoelastic properties of polymer surfaces investigated by nanoscale dynamic mechanical analysis. *Appl. Phys. Lett.* **88**, 131901 (2006).

5 Zhang, X. *et al.* Theoretical strength and rubber-like behaviour in micro-sized pyrolytic carbon. *Nat. Nanotechnol.* **14**, 762-769 (2019).

6 Haque, M. A. & Saif, M. In situ tensile testing of nanoscale freestanding thin films inside a transmission electron microscope. *J. Mater. Res.* **20**, 1769-1777 (2005).

7 Zandrini, T. *et al.* Effect of the resin viscosity on the writing properties of two-photon polymerization. *Opt. Mater. Express* **9**, 2601-2616 (2019).

8 Urbancová, P. *et al.* IP-Dip-based woodpile structures for VIS and NIR spectral range: complex PBG analysis. *Opt. Mater. Express* **9**, 4307-4317 (2019).

9 Lemma, E. D. *et al.* Mechanical properties tunability of three-dimensional polymeric structures in two-photon lithography. *IEEE Trans. Nanotechnol.* **16**, 23-31 (2016).

10 Frame, J. D., Green, N. G. & Fang, X. Modified Maxwell Garnett model for hysteresis in phase change materials. *Opt. Mater. Express* **8**, 1988-1996 (2018).

11 Radwin, R. G., Oh, S., Jensen, T. R. & Webster, J. G. External finger forces in submaximal five-finger static pinch prehension. *Ergonomics* **35**, 275-288 (1992).

12 Kong, D. & Xiao, X. High cycle-life shape memory polymer at high temperature. *Sci. Rep.* **6**, 33610 (2016).
